# Discovery and Computational Analyses of Novel Small Molecule Zika Virus Inhibitors

**DOI:** 10.3390/molecules24081465

**Published:** 2019-04-13

**Authors:** Siyu Zhu, Chaozai Zhang, Lina S. Huang, Xing-Quan Zhang, Yan Xu, Xiong Fang, Jiao Zhou, Meixian Wu, Robert T. Schooley, Ziwei Huang, Jing An

**Affiliations:** 1 School of Life Sciences, Tsinghua University, Beijing 100084, China; fx_09shuo@163.com; 2 Department of Medicine, Division of Infectious Diseases, School of Medicine, University of California San Diego, La Jolla, CA 92037, USA; chaozai224@126.com (C.Z.); lsh83@cornell.edu (L.S.H.); xiz002@ucsd.edu (X.-Q.Z.); m4wu@ucsd.edu (M.W.); rschooley@ucsd.edu (R.T.S.); 3 School of Pharmaceutical Sciences, Jilin University, Changchun 130021, China; 4 College of Arts and Sciences, Cornell University, Ithaca, NY 14853, USA; 5 School of Life and Health Sciences, Chinese University of Hong Kong, Shenzhen 518172, China; xyupstate@gmail.com; 6 Nobel Institute of Biomedicine, Zhuhai 519000, China; zhouj229@nobel-institute.org

**Keywords:** Zika virus, flaviviruses, structure-based virtual screening, non-structural protein 3, benzenediol, tetrahydroxy pentanoate

## Abstract

Zika virus (ZIKV), one of the flaviviruses, has attracted worldwide attention since its large epidemics around Brazil. Association of ZIKV infection with microcephaly and neurological problems such as Guillain–Barré syndrome has prompted intensive pathological investigations. However, there is still a long way to go on the discovery of effective anti-ZIKV therapeutics. In this study, an in silico screening of the National Cancer Institute (NCI) diversity set based on ZIKV NS3 helicase was performed using a molecular docking approach. Selected compounds with drug-like properties were subjected to cell-based antiviral assays resulting in the identification of two novel lead compounds (named Compounds **1** and **2**). They inhibited ZIKV infection with IC_50_ values at the micro-molar level (8.5 μM and 15.2 μM, respectively). Binding mode analysis, absolute binding free energy calculation, and structure–activity relationship studies of these two compounds revealed their possible interactions with ZIKV NS3 helicase, suggesting a mechanistic basis for further optimization. These two novel small molecules may represent new leads for the development of inhibitory drugs against ZIKV.

## 1. Introduction

The rapid spread of Zika virus (ZIKV) infection has become a severe and escalating threat to global health. ZIKV is a mosquito-borne pathogen and belongs to the genus Flavivirus of the family Flaviviridae that includes Hepatitis C virus (HCV), Dengue virus (DENV), and West Nile virus (WNV). ZIKV was first isolated in 1947 from a rhesus monkey around the Zika forest of Uganda [1]. However, it has not been paid much attention until 2015, when it rapidly remerged in at least 33 countries and territories [2,3], and became an expanding epidemic across Central and South America [4]. ZIKV infection can cause common symptoms such as fever, headache, arthralgia, conjunctivitis, as well as macular atrophy [5]. During 2015 and 2016, substantial evidence has confirmed its congenital, perinatal, and sexual transmission [6,7], and noted that ZIKV infection is directly associated with frightening neural diseases including Guillain–Barré syndrome [8] and congenital microcephaly [9]. ZIKV can be transmitted from a mother to a developing fetus during pregnancy and therefore inflict severe birth defect including fetal growth restriction, neurological and ocular abnormalities, or even perinatal death [10]. This fast-emerging pandemic and the related severe clinical situations have invigorated efforts to seek effective antiviral therapeutics against ZIKV for both prevention and post-infection treatment.

ZIKV has a positive-sense single-stranded genomic RNA that encodes a single polyprotein precursor consisting of both structural proteins and non-structural proteins (NSPs) from NS1 to NS5. Three-dimensional structures of ZIKV NSPs including NS1 [11,12], NS2B-NS3 protease [13,14,15], NS3 helicase [16], NS5 methyltransferase [17,18,19], as well as NS5 RNA polymerase have been determined consecutively [20]. They are cooperatively involved in viral polyprotein processing and RNA synthesizing processes including RNA maturation, splicing, as well as nuclear export [21]. A major component involved in the ZIKV replication complex is NS3, a multifunctional protein with an *N*-terminal serine protease domain (NS3Pro) responsible for polyprotein processing, and a C-terminal region that serves as an RNA helicase (NS3Hel) for viral replication [16,20]. ZIKV protease is a two-component protease consisting of the *N*-terminal region of NS3 and the cytoplasmic region of NS2B as its cofactor; they collaborate to position the catalytic triad to catalyze its substrate [22]. ZIKV NS3Hel belongs to the helicase superfamily of nucleic acid-dependent NTPases and are capable of unwinding DNA or RNA duplex substrates [23]. The crucial function of NS3Hel during viral replication has made it a promising drug target for direct-acting antiviral agents (DAA) [24].

Discovery of a novel drug for antiviral therapy is often a time- and labor-consuming process [25,26]. To date, there are still very few successful cases in the development of clinically approved treatment therapies for Zika virus infection, making the discovery of anti-ZIKV drugs an urgent task [4]. In recent years, structure-based virtual (also known as in silico) screening has become a useful approach for identifying potential lead compounds [27]. In silico screening is considered to be one of the rapid, efficient, and cost-effective methods for screening a large set of compounds [28]. Such an approach has been proven to be valuable in antiviral DAA discovery [29,30]. Indications from the development of DAAs for other flaviviruses including DENV and WNV emphasized the strategy of blocking viral enzymes [29,30]. Recent structural evidence has revealed molecular mechanisms of NS3 helicase activity and accordingly provided precise information for the rational drug design of small molecule inhibitors against ZIKV [14,16,22,31]. In this study, structure-based virtual screening on ZIKV NS3Hel followed by antiviral biological assays was performed with the aim of discovering novel inhibitors. 

## 2. Results 

### 2.1. Structure-Based Virtual Screening of a Diverse Library

**Figure 1:**
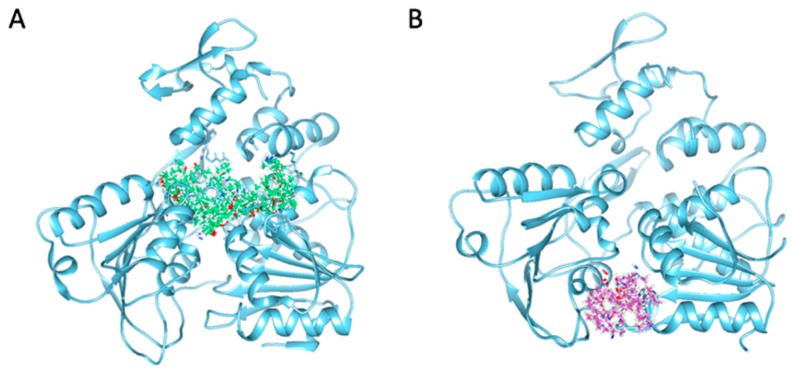
Molecular demonstration of the docking modes generated according to the binding grooves of (**A**) single-stranded RNA (ssRNA) (protein data bank (PDB) ID: 5GJB, shown in spring green) and (**B**) nucleoside triphosphate (NTP) (PDB ID: 5GJC, shown in pink within Zika virus (ZIKV) non-structural protein 3 helicase (NS3Hel) (shown in sky blue).

Crystal structures of ZIKV NS3 helicase in complex with single-stranded RNA (ssRNA) and nucleoside triphosphate (NTP), respectively, have unveiled a molecular basis for RNA unwinding activities [16,32,33]. ssRNA runs across the central groove of each domain with the bases stacked against each other, while NTP binds the cleft between domains 1 and 2 [16,32,33]. Here, we performed a virtual screening by docking small molecules from a compound library to ZIKV NS3Hel active sites including both RNA and NTP binding cavities (Figure 1). The NCI diversity compound dataset was derived from almost 250,000 compounds and the final set was selected using the programs Chem-X (Oxford Molecular Group) and Catalyst (Accelrys, Inc., San Diego, CA, USA) using defined pharmacophoric centers and distance intervals. A total of 1974 compounds with structural diversity were used to perform the docking study. Docking scores of all compounds were presented by SYBYL and 5% top-scoring compounds with a C_Score > 4.0 and a crash value > −2.0 were ruled out. Most of them revealed interaction with three to five residues around the pocket primarily by forming hydrogen bonds with both main and side chains on these amino acids. Further, a drug-likeness filter was set as molecular weight from 200 to 500 g/mol, Log-*P* value from −2.00 to 5.00, number of hydrogen acceptors from 2 to 8, and donors from 0 to 5. Compounds in accordance with this filter were ordered from NCI library of the Developmental Therapeutics Program of NCI/NIH.

### 2.2. Antiviral Activity Against in Vitro ZIKV Infection

**Figure 2:**
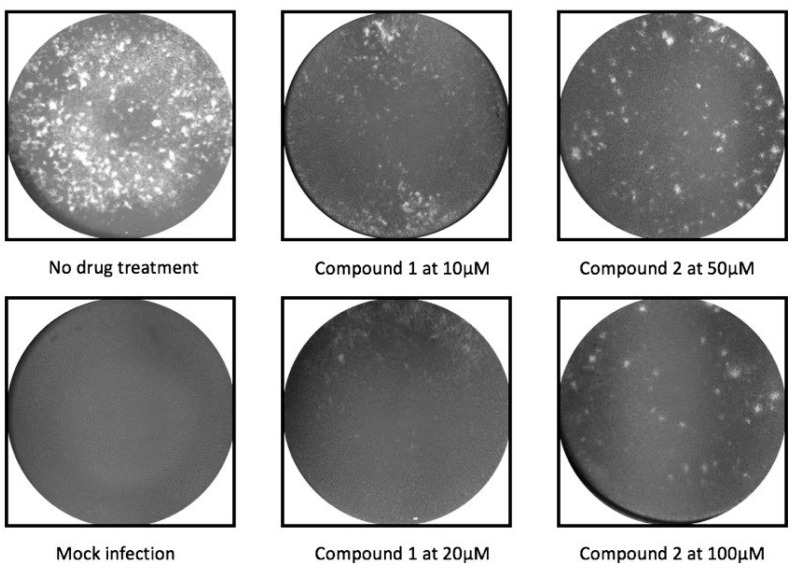
Reduction of plaque formation by Compound **1** at 10 and 20 μM and Compound **2** at 50 and 100 μM. Plaque formation developed on BHK-21 cell lines by ZIKV infection with no drug treatment served as a negative control.

**Figure 3:**
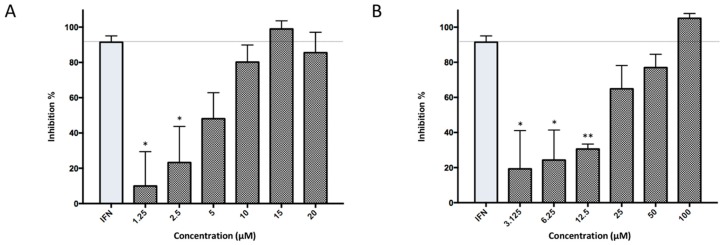
Dose-dependent inhibitory activities of Compounds **1** and **2**. (**A**) Inhibitory activities of Compound **1** under a gradient concentration from 1.25 μM to 20 μM against ZIKV infection on BHK-21 cell lines. (**B**) Inhibitory activities of Compound **2** under a gradient concentration from 3.125 μM to 100 μM against ZIKV infection on BHK-21 cell lines.

**Figure 4:**
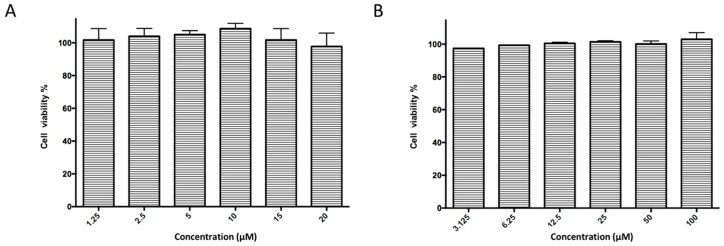
Examination of any cytotoxicity of Compounds **1** and **2**. Percent cellular viability was measured for (**A**) Compound **1** under a gradient concentration from 1.25 μM to 20 μM. (**B**) Compound **2** under a gradient concentration from 3.125 μM to 100 μM on BHK-21 cell lines.

Subsequently, ordered compounds were tested on BHK-21 cell lines to determine their inhibitory potency against ZIKV infection. Eight of them (Table 1), designated as Compounds **1**–**8** (NSC10580, ZINC01706300; NSC45741, ZINC263598830; NSC99676, ZINC100132692; NSC95910, ZINC1621537; NSC20172, ZINC2046417; NSC100297, ZINC00001260; NSC299209, ZINC1871679; NSC99799, ZINC2291012), assisted cell survival against viral infection as shown by cytopathic effect inhibition assay. They showed inhibitory activities of more than 20% at the concentration of 20 μM. Among these compounds tested, Compound **1** (NSC10580, ZINC01706300)—an amphipathic benzenediol structure—and Compound **2** (NSC45741, ZINC263598830)—a highly hydrophilic molecule—exhibited more than 50% inhibition on BHK-21 cell lines against ZIKV infection. Thus, these two compounds were selected as the leads for further investigation. We assessed their ability to reduce plaque formation during ZIKV in vitro infection. In the presence of Compound **1** or Compound **2**, plaque formation of ZIKV was markedly reduced compared to the virus control group in the absence of drugs (Figure 2). Compound **1** at the concentration of 20 μM and Compound **2** at the concentration of 100 μM achieved significant plaque reduction. We further determined their IC_50_ values against ZIKV infection. Both of them dose-dependently suppressed ZIKV-induced cytopathic effects, with IC_50_ values at a micro-molar level (8.5 μM and 15.2 μM, respectively) (Figure 3). IFN-α at 20 IU served as a positive control in all antiviral tests. Cytotoxicity studies of Compounds **1** and **2** were further performed to confirm that there was no obvious toxicity on BHK-21 cell lines compared to DMSO-treated cells within the above dose range (Figure 4).

### 2.3. Potential Inhibition on Viral Replication Process

**Figure 5:**
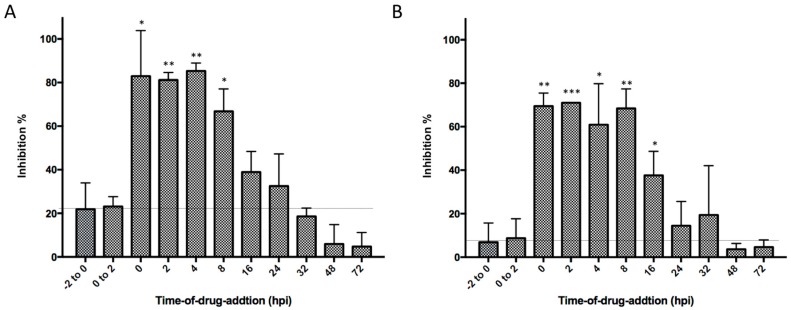
Time-of-drug-addition assay of Compounds **1** and **2**. (**A**) Inhibitory activities while treated with Compound **1** under a concentration of 20 μM at various time points (from −2 to 0 h post infection (hpi), from 0 to 2 hpi, at 0, 4, 8, 16, 24, 32, 48, and 72 hpi, respectively). (**B**) Inhibitory activities while treated with Compound **2** under a concentration of 50 μM at the above time points.

To further explore the inhibitory mechanism, a time-of-drug-addition experiment from pre-treatment to 72 h post infection (hpi) was performed to investigate whether our hit compounds blocked the stage of viral binding, entry, or replication during the infection cycle. Compound **1** and Compound **2** exerted high antiviral activities when administered during the first 8 h after virus addition, while less or almost no suppression of viral activity was observed for either pre-treatment or treatment from 0 hpi to 2 hpi (Figure 5). Both of two compounds started to lose their antiviral potency when added after 16 hpi. These data indicated that both Compound **1** and Compound **2** had the potential to block viral replication process instead of viral binding or entry. 

### 2.4. Binding Modes and Interactions with NS3 in Silico

**Figure 6:**
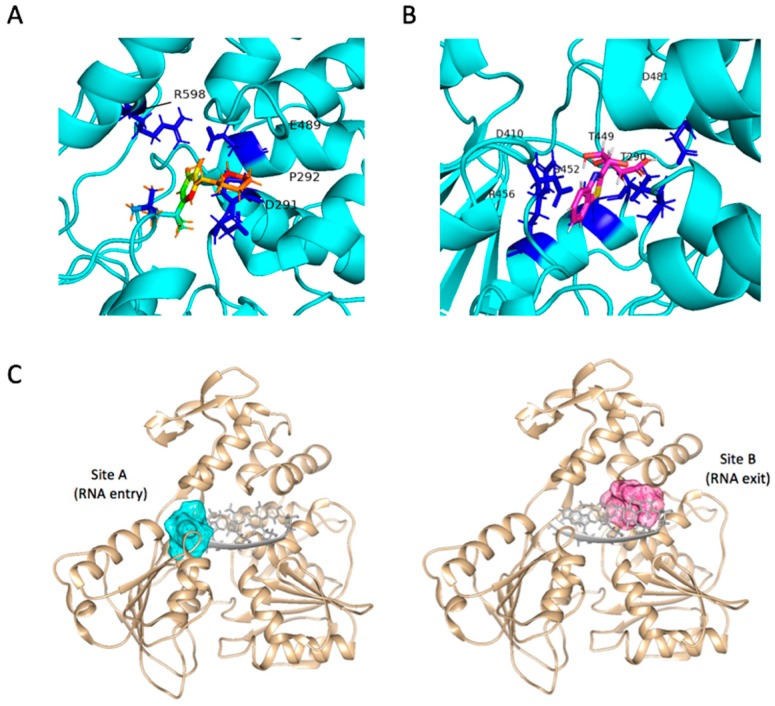
(**A**) Predicted binding modes of Compound **1** (shown in green with color by atom type) inside ZIKV NS3Hel pocket with crucial interacting residues (shown in blue). (**B**) Predicted binding modes of Compound **2** (shown in magenta with color by atom type) inside ZIKV NS3Hel pocket with crucial interacting residues (shown in blue). (**C**) Molecular demonstration of target site A and site B (shown in cyan and pink, respectively) generated according to predicted interacting residues and relative locations to ssRNA (shown in gray) inside ZIKV NS3Hel (shown in tan), corresponding to RNA entry and exit path.

To understand potential modes of action for their inhibitory activities on ZIKV, we scrutinized the in silico binding modes of these two compounds. Both Compounds **1** and **2** were docked into NS3Hel pockets with favorable C_Scores and crash values and showed interaction with residues around the RNA binding cavity, indicating their potential to block the RNA unwinding reaction. Markedly, though both were predicted to block NS3Hel, two hit molecules demonstrated distinctive binding patterns inside the pocket. Compound **1** fulfilled the pockets near the interface of domains 1 and 3. Hydroxyl groups on 1,2-benzenediol formed hydrogen bonds with side chains of Glu489 and Arg598 in opposite directions, while the nitrogen on the hexatomic ring projecting towards the side chain of Asp291. Piperidine ring on Compound **1** might act as a hydrophobic core by interacting with Pro292 (Figure 6A). Compound **2** was docked into the site within domain 2, with its side-chain hydroxyl groups forming hydrogen bonds with Thr290, Asp410, Ser452, and Arg456. The terminal carboxyl was surrounded by Thr449 and Asp481, therefore interrupting the original salt bridge and hydrogen bond between that is essential for RNA recognition and binding (Figure 6B). The two distinctive sites, designated Site A and Site B (Figure 6C), located respectively within the two clefts—RNA entrance crevice and RNA exit crevice. They are both required for RNA unwinding [16,34]. Crystal structures of Zika virus helicase in recognizing its substrates indicated that residues Asp410 and Arg598 could form hydrogen bonds with its substrate [32]. In particular, side chain of Asp410 in the inner core could form a salt bridge with residue Lys431 that is crucial for the binding of N3 atom of the adenine base. Its carbonyl oxygen also contributes multiple hydrogen bonds with the 2′-OH moieties from the ssRNA. Molecular docking results of Compounds **1** and **2** demonstrated highly competitive binding modes against the substrate binding site.

### 2.5. Absolute Binding Free Energy Calculation

**Figure 7:**
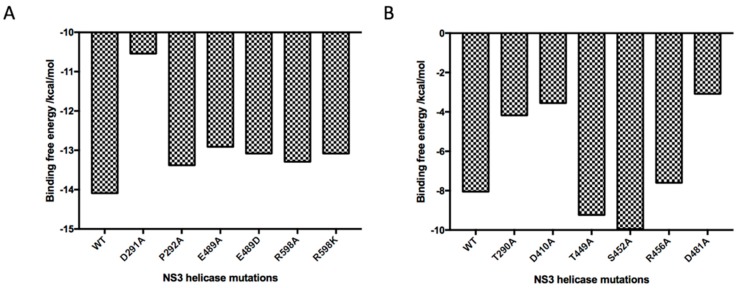
Absolute binding free energy values for two ligand–protein docking complexes using PDLD/S-LRA/β methodology. All energies are presented in Kcal/mol. (**A**) Calculated free energy of Compound **1** to NS3 helicase Wildtype (WT) and mutants (D291A, P292A, E489A, E489D, R598A, and R598K). (**B**) Calculated free energy of Compound **2** to NS3 helicase Wildtype (WT) and mutants (T290A, D410A, T449A, S452A, R456A, and D481A).

Encouraged by our docking models, microscopic free energy calculation together with in silico mutagenesis of residues that were observed in the binding modes was performed to further validate the hypothesis of above molecular interactions with NS3 helicase (Figure 7). The binding free energy of Compound **1** was dramatically reduced by site mutation of Asp291 since the difference of Gibbs free energy corresponds to exponential changes in dissociation constant (K_D_) values, indicating the relatively strong interaction between the hexatomic ring of Compound **1** and the side chain of Asp291. Mutations on charged residues Glu489 and Arg598, as well as uncharged residue Pro292, also essentially affected the binding of Compound **1**, consistent with the above docking analysis. For Compound **2**, mutations on Thr290, Asp410, Arg456, and Asp481 reduced the absolute values of binding free energy in various degrees, demonstrating that these residues significantly contributed to ligand binding while residues including Thr449 and Ser452 had relatively less importance for affinity. This could, as well, be caused by conformational changes of the complex structure during molecular dynamics. The resulting absolute binding free energy also showed that Compound **1**, in agreement with antiviral experiments, had a higher affinity (−14.09 Kcal/mol) than Compound **2** (−8.05 Kcal/mol) to NS3 helicase. These theoretical calculations provided us quantitative evidence to support our experimental and docking data and inspiring insights to discuss ligand-target binding details.

### 2.6. Ligand-Based Search and Structure-Activity Relationship Analysis of Derivatives

**Figure 8:**
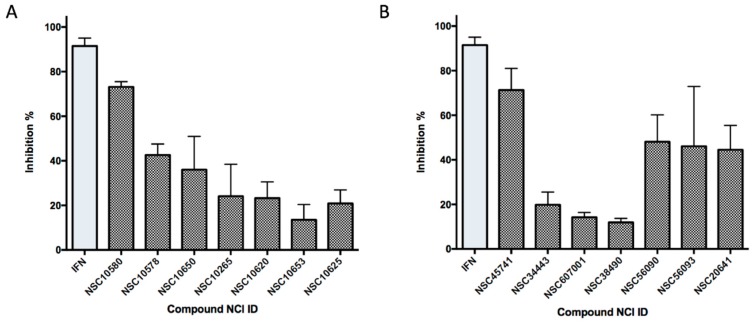
Inhibitory activities of derivative compounds against ZIKV infection on BHK-21 cell lines. (**A**) Inhibitory activities of Compounds **1** and **1A**–**1F** at a concentration of 20 μM. (**B**) Inhibitory activities of Compounds **2** and **2A**–**2F** at a concentration of 50 μM. IFN-α at 20 IU served as a positive control.

On the basis of the above antiviral activities and favorable binding modes, small-molecule compounds with scaffolds similar to those of Compound **1** and Compound **2** were examined for further structure-activity relationship (SAR) analysis (Table 2). Analogs available from the NCI compound database were ordered and evaluated by in vitro assay accordingly (Figure 8). Compound **1A,** bearing a morpholine instead of piperidine, exhibited a decrease in antiviral activity, indicating the role of the hydrophobic core in Compound **1** in its interaction with residue Pro292. Deletion of a phenolic hydroxyl group, presented in Compound **1B**, or replacing it by alkyl chain, presented in Compound **1C**, reduced the inhibitory activity as well, which was consistent with the potential interaction with residue Arg598 shown in the above analysis. Compounds **1D**–**F** further indicated that either replacing piperidine by hydrophilic groups or shortening the alkyl chain resulted in a decrease in antiviral activity. For Compound **2**, replacing the terminal carboxyl group with an alcoholic hydroxyl group in Compound **2A** led to a lower activity. Further depletion or displacement of acidic groups, shown in Compounds **2B** and **2C**, also demonstrated the interaction of those acidic chains with the protein residues including Threonine, Arginine, and Aspartic acids as described above. Compounds **2D** and **2E** indicated that substitution of benzothiazole by benzimidazole might help increase the inhibitory effect. Based on Compound **2E**, we further found that introducing other hydrophobic groups such as hexamethylene (Compound **2F**) could also partly maintain the antiviral activity as was expected. The above SAR analysis might shed light on further studies of binding patterns and modifications for these two antiviral leads.

## 3. Discussion

Increasing popularity of global travel, as well as the wider spread of mosquito vectors due to the climate change, has nowadays accelerated the transmission of flaviviruses including ZIKV. Its severe damage on both fetal and adult neural systems urged us to understand the pathological mechanism and find solution to block its infection. However, despite the urgency of fighting ZIKV, no vaccines or antiviral therapeutics have been approved clinically for combating ZIKV infection. Enzymatic proteins involved in viral replication and assembly have long been considered as crucial drug targets to develop therapeutic agents. Since 2015, several studies have reported results of high-throughput screening of compound libraries to find small molecules active against ZIKV infection using either a whole-virus assay [35,36] or protein-based assays [37,38,39]. A couple of hits or potential lead molecules were described, whereas others failed to act effectively against viral infection through exhibiting binding or inhibitory activity on the target enzymes [37].

Structural homology of ZIKV enzymatic proteins with those of other flaviviruses prompted the interest in previously reported molecules showing inhibition on HCV, DENV, WNV, and Japanese Encephalitis Virus (JEV). Efforts were made to retest a portion of them, including protease inhibitors [37,39,40] and RNA-dependent RNA polymerase inhibitors [38], to examine their potential effects on ZIKV. Among the compounds tested, a couple of molecules could act against ZIKV with IC50 values at a micro-molar level. Most of them, however, did not show an equal inhibitory activity to their original target enzyme from HCV or other flaviviruses, which is understandable considering the conformational flexibility, exposure to solvent, and slight sequential differences among these viral proteins. Moreover, unlike the protease, few specific NS3 helicase/NTPase inhibitors have been reported for flaviviruses [41,42,43]. So far, none of these flaviviral helicase inhibitors was efficaciously repurposed to block ZIKV helicase. Therefore, our attention has been basically focused on this underexploited target. Structural characterizations of ZIKV NS3Hel have been accomplished since 2016 [13,14,16,32,33], providing insightful structural basis for rational drug design. Compared with those NS3Hel from other flaviviruses, structures of ZIKV NS3Hel disclosed a similar core scaffold but variable loops—the *P* loop (residues 196–203) and the RNA-binding loop (residues 244–255) [16], involved correspondingly in the interaction of NS3Hel with 7-mer RNA and NTP. This underlined the necessity of finding novel inhibitors for ZIKV NS3Hel.

Our drug discovery strategy here is a combinational workflow of virtual screening and antiviral assessment to facilitate the discovering process of novel leads specifically targeting ZIKV. Previous efforts on virtual screening of ZIKV inhibitors, to the best of our knowledge, have been focused on NS2B-NS3 protease [44,45] and NS5 methyltransferase and RNA-dependent RNA polymerase [44,46,47]. Most of these studies were based on homology modeling instead of crystal structures or were not evaluated by antiviral assays. Here, we selected a database of diverse chemical structures and two proven ligand-binding sites on ZIKV NS3Hel crystal structures with the aim of minimizing the screening time and effort. We then assessed antiviral activity of best-docked molecules on a whole-virus assay and found eight compounds exhibited anti-ZIKV potency. The two most potent compounds were further selected as our lead compounds. Both of the two compounds dose-dependently inhibited ZIKV infection in vitro, and therefore had potential for further in vivo and clinical studies. Besides the determination of their antiviral activities and exploration of possible inhibitory mechanisms, a series of computational analyses including molecular dynamics simulation, absolute binding free energy calculation, and SAR analysis were performed to elucidate the potential mechanism of binding and inhibition.

Compound **1** is an amphipathic molecule bearing a benzenediol scaffold. Computer docking simulations predicted the interaction with three polar residues of ZIKV NS3Hel and suggested its possible blockade of the entrance cleft of RNA binding. Compound **2** exhibited a hydrophilic property with a tetrahydroxy pentanoate structure. Predicted binding model indicted its potential to block the exit cleft of RNA binding. Compound **2** was previously tested in a study of TRAIL pathway-specific anticancer agents, yet no further biological application has been reported before [48].

Meanwhile, drug development is a long-term process involving significant effort, time, and expense. Therefore, drug repurposing has been considered a viable, cost-effective, and efficient way to speed up clinical application. It should be noted that Compound **7** (NSC100297, ZINC00001260) reported here is a commercially used product, Dimetacrine, as an anti-depressive drug. It serves as an antagonist targeting Acetylcholinesterase and reduces serine hydrolase activity [49,50]. In addition, Compound **8** (NSC99799, ZINC22910125) is known to the market as Papaveroline, an alkaloid found in opium. While the mechanism of pharmacological actions remains mostly unidentified, studies have confirmed its inhibition on phosphodiesterase [51]. It remains to be seen whether further studies might reveal any potential value of these existing drugs in a possible new use for treating ZIKV.

## 4. Conclusions

Wide spread of ZIKV and the associated severe neurological damage have prompted intensive efforts to discover effective antiviral agents to fight this pandemic. With the aim of discovering novel chemical structures targeting ZIKV infection, in this study we carried out virtual screening, antiviral assays, and computational analyses to successfully identify two novel small molecules with anti-ZIKV IC_50_ values of less than 20 μM. These two different chemical scaffolds are reported for the first time, to our knowledge, to have anti-ZIKV activity and may represent promising leads for developing anti-ZIKV agents. Compound **1** and Compound **2** showed competitive antiviral activity with IC_50_ values of 8.5 μM and 15.2 μM, respectively, and displayed specific inhibition against viral replication processes. Predicted binding modes implied their potential to block the activity of NS3 RNA helicase and lead to further investigation and optimization. Based on biological data and computational insights, our study discovered two lead molecules with anti-ZIKV activity, paving the way for future development of clinical therapeutics against ZIKV infection.

## 5. Materials and Methods

### 5.1. Hardware and Software

In silico studies were performed on Dell Workstation Precision 5810 with 2E5-2600 V3 2.133 GHz processor, 6 GB RAM, and 256 GB hard drive running in Red Hat Enterprise Linux (RHEL) operating system. Bioinformatics software SYBYL-X 2.1.1 (Tripos Associates, St. Louis, MO, USA) was used for virtual screening and docking simulations. MOLARIS package version 9.15 (USC) was used for molecular dynamics simulations and binding free energy calculations. Chimera (UCSF) and PyMol Molecular Graphics software (Schrödinger) helped analyze details of protein–ligand interactions. Online resources including protein data bank (http://www.rcsb.org/pdb/) and Enhanced National Cancer Institute (NCI) database (https://cactus.nci.nih.gov/ncidb2.2/) were utilized to perform this study.

### 5.2. Protein Refinement and Compound Database Preparation

Protein data bank (PDB) files (PDB ID: 5GJB, 1.7 Å X-ray resolution; PDB ID: 5GJC, 2.2 Å X-ray resolution; PDB ID: 5JMT, 1.8 Å X-ray resolution) of ZIKV NS3 crystal structures were retrieved from PDB database. All water molecules were removed and polar hydrogen atoms were assigned. After that, protein structures were analyzed using the Protein Structure Preparation Tool in SYBYL-X 2.1.1. Explicit hydrogen, disulfide bonds, and charges were assigned wherever missing, and side-chain amides were fixed. NCI diversity set was downloaded from NCI compound database. 3D structures were generated by SYBYL-X 2.1.1 and essential hydrogen atoms were added. Molecular minimization was applied with the Gasteiger–Huckel charge and a distance-dependent dielectric function (dielectric constant = 1.00), energy gradient of 0.001 Kcal/mol and maximum iterations of 2000 of Merck Molecular Force Field 94 (MMFF94) method.

### 5.3. Virtual Screening using Surflex-Dock

Molecular docking studies were carried out on SYBYL-X 2.1.1 to screen potential binding candidate compounds. Surflex-Dock (SFXC) algorithm serves as an automatic molecular docking program using an empirical scoring function and a patented searching engine [52]. A negative-phase “protomol” with a threshold of 0.50 and bloat set to 0 Å was generated for each protein structure. During docking process, the maximum number of poses per ligand was set to 20. Other parameters were established using default values. After docking, minimized ligand poses and their rankings were returned. Consensus scores (C_Scores), representing an overall of Dock_Scores, PMF_Scores, Gold_Scores, and Chem_Scores were provided for ranking the binding affinity of ligands to the receptor (SYBYL C_Score module = Dock_Score + PMF_Score + Gold_Score + Chem_Score). Crash represented the degree of inappropriate penetration into the protein as well as self-clashing of ligands.

### 5.4. In silico Molecular Interaction Analysis

Predicted binding poses of in vitro validated compounds were implemented using flexible protein–ligand docking simulations on SYBYL-X 2.1.1 program. Hit compounds were docked to the binding pockets with an acceptable target flexibility. Hydrogen and heavy atoms around binding sites of protein target were able to change molecular coordinates. Amino acid interactions of the docked molecules were analyzed by Chimera and PyMol.

### 5.5. Molecular Dynamics Simulation

Molecular dynamics (MD) simulation of docking complexes was performed using polarizable ENZYMIX force field in MOLARIS package version 9.15. All MD simulations were completed using spherical boundary conditions with a sphere of 18 Å radius solvated with water molecules subject to the standard MOLARIS surface-constraint all-atom solvent (SCAAS) boundary conditions and the local reaction field (LRF) long-range treatment, surrounded by 2 Å radius with Langevin dipole surface embedded in a bulk continuum. The C atom near the geometric center of the ligand is defined as the center of the simulations sphere. Each structure was relaxed by 3.0 ns MD simulation by being gradually heated to a target temperature of 300 K with a step size of 1.0 fs.

### 5.6. Ab Initio Binding Free Energy Calculation

Binding free energy calculations were performed using the POLARIS module in MOLARIS package version 9.15 [16]. All binding free energies were calculated using the Protein Dipole Langevin Dipole (PDLD) method within its semimacroscopic-linear response approximation (PDLD/S-LRA) with the LIE nonpolar term (PDLD/S-LRA/β). The MD runs were performed with the SCAAS spherical boundary condition and the local reaction field (LRF) long-range treatment [16]. The PDLD/S-LRA calculations involved a replacement of the SCAAS water molecules by Langevin Dipoles and the results were averaged over 20 configurations. The simulations were performed following the previously reported procedure including relaxation dynamics of 90.0 ps, dynamics in water for 24.0 ps, and dynamics in the protein for 24.0 ps with a step size of 1.0 fs [16].

$$\Delta G_{bind}^{PDLD/S-LRA/\beta }=\Delta G_{bind}^{elec/PDLD/S-LRA}+\beta [{\langle U_{vdw,l}^{p}\rangle }_{l}-{\langle U_{vdw,l}^{w}\rangle }_{l}]$$

### 5.7. Cell Culture and Compounds Preparation

BHK-21 cells (Baby Hamster Syrian Kidney-21) and Vero cells (African Green Monkey kidney) were recovered and cultured using MEM Alpha Medium (HyClone, Logan, UT, USA) supplemented with 10% fetal bovine serum (FBS) (Gibco, Grand Island, NY, USA), 100 IU penicillin, 0.1 mg/mL streptomycin, and 1% HEPES at 37 °C in a 5% CO_2_ humidified environment. Compounds selected from the top-scoring list were ordered from NCI compound library. All compounds in powder were dissolved in DMSO as 10mM stock solutions, analyzed by NMR and mass spectrometry, and stored at −80 °C for further dilution.

### 5.8. Virus Preparation and in Vitro Infection Assay

A Cambodian clinical ZIKA virus strain FSS13025 (GenBank: KU955593.1) was originally obtained from a 3-year-old patient in 2010 [53]. Original viral stocks were then amplified in Vero cell lines and titrated using quantitative RT-PCR and plaque assays. For infections, freshly cultured Vero or BHK-21 cells were diluted into 1 × 10^6^ cells/mL with PBS buffer. Then, 24-well flat-bottomed plates were incubated at 37 °C in 5% CO_2_ and one milliliter of cell solutions was inoculated to each well and cultured overnight with culture medium. After that, cells were infected by ZIKV at a MOI of 0.01 for 2 h. For mock infections, an equal volume of culture medium was used. Cells were incubated at 37 °C in 5% CO_2_ for 72–120 h after infection.

### 5.9. Virus Plaque Reduction Assay

BHK-21 cells were diluted into 1 × 10^6^ cells/mL and cultured overnight with BHK-21 medium as described above. ZIKV at a MOI of 0.01 was added to each well and infected BHK-21 cells for 2 h. For mock infections, an equal volume of culture medium was used. After that, cells were infected by ZIKV at a MOI of 0.01 for 2 h. For mock infections, an equal volume of culture medium was used. After that, cells were washed by PBS twice and 1 mL of fresh medium was added respectively with each candidate antiviral compound at concentrations of 10 and 20 μM for Compound **1** and 50 and 100 μM for Compound **2**. Cells were incubated at 37 °C in 5% CO_2_ for 120 h after infection. To fix the cells, 10% formaldehyde solution was added to each well for 30 minutes. After fixing, formaldehyde was discarded, and the cells were covered with a minimal amount of crystal violet solution for 1.5 h. Then, crystal violet stain was gently washed off with water. The plaques were presented for further analysis. The percentage of plaque inhibition to the virus control group of each compound was evaluated: Inhibitory activity = (1 − number of plaques produced with candidate compounds/number of plaques produced without candidate compounds) %.

### 5.10. Cytopathic Effect Inhibition Assay

The IC_50_ values of primary hit compounds were determined by cytopathic effect inhibition assay. BHK-21 cells were cultured and infected as described above. Each primary hit antiviral compound was added at a gradient concentration. IFN-α at a concentration of 20 IU served as positive control. After 120 h, inhibition of cytopathic effects was assessed using a Cell Titer 96^®^ Cell Proliferation Assay (Promega Corporation, WI, USA) according to manufacturer’s instructions. Cell Titer-Blue reagent was added and incubated for 2 h while viable cells reduced resazurin into resorufin. The amount of resorufin produced was determined at λ_excitation_ 560 nm and λ_emission_ 590 nm fluorescence determination using a spectrophotometric microplate reader (Bio-Rad, Hercules, CA, USA).

### 5.11. Time-of-Drug-Addition Assay

Time-of-addition experiment was conducted to explore which stage of the viral life cycle is affected by hit compounds. BHK-21 cells were cultured and infected as described above. Compound **1** at a concentration of 20 μM and Compound **2** at a concentration of 50 μM and was respectively added to the assay medium at 2 h prior to infection (pre-treatment), at 0 (at the time of infection) to 2 hpi, or at 0, 2, 4, 8, 16, 32, 48, and 72 hpi. At 120 hpi, inhibition of cytopathic effects was assessed using a Cell Titer 96^®^ Cell Proliferation Assay as described above.

### 5.12. Cytotoxicity Assay

Cytotoxicity of lead compounds against BHK-21, SupT-1 (Human lymphoma) as well as HEK293T (Human embryonic kidney) cell lines were evaluated by Cell Titer-Blue viability assay as well. BHK-21 cells were seeded in a 96-well plate and cultured as described above. SupT-1 and HEK293T cells were cultured respectively in RPMI-1640 Medium and Dulbecco’s Modified Eagle’s Medium (DMEM) instead of MEM Alpha Medium. Compound solutions under a gradient concentration same as the inhibition assay were added. Cells were incubated for 120 h, followed by addition of Cell Titer-Blue reagent to each well and further incubated for 2 h according to manufacturer’s instructions. Determination of cell viability was the same as described above.

### 5.13. Statistical Analysis

Biological assays were performed in triplicate and data were analyzed in Microsoft Excel and plotted in GraphPad Prism 7 (GraphPad Software Inc., CA, USA). Average values were expressed as mean ± SD or SEM, n ≥ 3. The results of Virus plaque reduction assay were representatives of three independent experiments. A *p*-value less than 0.05 was considered statistically significant. * *p*-value < 0.05, ** *p*-value < 0.005, *** *p*-value < 0.0005. 

## Figures and Tables

**Table 1:** Initial compounds identified by structure-based virtual screening and antiviral evaluation. Compounds presented by National Cancer Institute (NCI) ID, ZINC ID, molecular name, 2D chemical structure, chemical properties, and experimental inhibitory activities. Inhibitory activities at 20 μM were evaluated by in vitro ZIKV infection assay.

Compound No	NCI ID, ZINC ID and Molecular Name	Structure	Molecular Weight (g/mol)	Log-*P* Value	H-Bond Acceptor Count	H-Bond Donor Count	Inhibitory Activities %
**1**	NSC10580 ZINC01706300 3-(1-piperidinylmethyl)-5-(1,1,3,3-tetramethylbutyl)-1,2-benzenediol	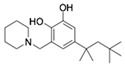	319.489	4.798	3	3	73.241 ± 3.336
**2**	NSC45741 ZINC263598830 5-(1,3-benzothiazol-2-yl)-2,3,4,5-tetrahydroxypentanoate	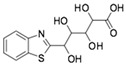	321.279	−0.503	8	4	54.475 ± 14.460
**3**	NSC99676 ZINC100132692 1,3-dimethyl-8-(3-phenylpropylsulfanyl)-6-sulfanylidene-7*H*-purin-2-one	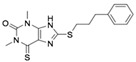	346.467	3.054	4	1	39.024 ± 4.243
**4**	NSC95910 ZINC1621537 8-((benzylthio)methyl)-1,3-dimethyl-3,9-dihydro-1*H*-purine-2,6-dione	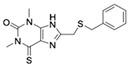	316.386	1.394	6	1	34.920 ± 14.255
**5**	NSC20172 ZINC2046417 *N*-acetyl-3-(1-naphthyl) alanine	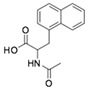	257.288	1.972	3	2	30.965 ± 4.292
**6**	NSC100297 ZINC00001260 3-(9,9-dimethylacridin-10-yl)-*N*,*N*-dimethylpropan-1-amine	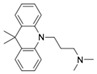	294.210	4.260	2	0	25.455 ± 2.185
**7**	NSC299209 ZINC1871679 4-((3-quinolinylmethyl) amino) benzenesulfonamide	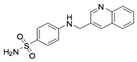	313.373	2.494	5	2	21.595 ± 0.573
**8**	NSC99799 ZINC22910125 1-(3,4-dihydroxybenzyl)-6,7-isoquinolinediol	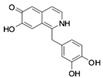	283.283	2.648	4	5	20.080 ± 0.113

**Table 2:** Derivative compounds identified by ligand-based search for SAR analysis and antiviral evaluation. Derivative compounds presented by NCI ID, ZINC ID, molecular name, 2D chemical structure, chemical properties, and experimental inhibitory activities. Inhibitory activities at 20 μM (for Compound **1** and derivatives) and 50 μM (for Compound **2** and derivatives) were evaluated by in vitro ZIKV infection assay.

Compound No	NCI ID, ZINC ID and Molecular Name	Structure	Molecular Weight (g/mol)	Log-*P* Value	H-bond Acceptor Count	H-bond Donor Count	Inhibitory Activities %
**1**	NSC10580 ZINC01706300 3-(1-piperidinylmethyl)-5-(1,1,3,3-tetramethylbutyl)-1,2-benzenediol	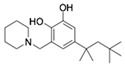	319.489	4.798	3	3	73.161 ± 2.363
**1A**	NSC10578 ZINC31613795 3-(morpholin-4-ylmethyl)-5-(2,4,4-trimethylpentan-2-yl)benzene-1,2-diol	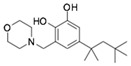	321.461	3.644	4	2	42.602 ± 4.906
**1B**	NSC10650 ZINC1706378 2-(piperidin-1-ylmethyl)-4-(2,4,4-trimethylpentan-2-yl)phenol	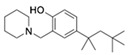	303.490	5.092	2	1	36.027 ± 14.915
**1C**	NSC100265 ZINC1662241 2,4-bis(2-methylbutan-2-yl)-6-(piperidin-1-ylmethyl)phenol	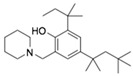	331.544	5.753	2	1	24.118 ± 14.307
**1D**	NSC10620 ZINC1706337 2-[[bis(2-hydroxyethyl)amino]methyl]-4-tert-butylphenol	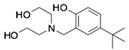	267.369	1.476	4	3	23.266 ± 7.250
**1E**	NSC10653 ZINC1706381 2,6-bis[(dimethylamino)methyl]-4-(2,4,4-trimethylpentan-2-yl)phenol	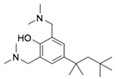	320.521	4.229	3	1	20.885 ± 6.033
**1F**	NSC10625 ZINC610623 4-tert-butyl-2,6-bis[(dimethylamino)methyl]phenol	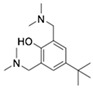	264.413	2.813	3	1	13.538 ± 6.838
**2**	NSC45741 ZINC263598830 5-(1,3-benzothiazol-2-yl)-2,3,4,5-tetrahydroxypentanoate	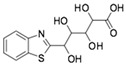	321.279	−0.503	8	4	71.316 ± 9.711
**2A**	NSC34443 ZINC4777682 1-(1,3-benzothiazol-2-yl)pentane-1,2,3,4,5-pentol	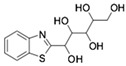	285.314	−0.595	7	5	19.868 ± 5.688
**2B**	NSC607001 ZINC1609998 1-(benzo[d]thiazol-2-yl)-5-hydroxypentan-1-one	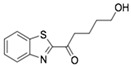	235.301	2.642	4	1	14.238 ± 2.179
**2C**	NSC38490 ZINC1670696 Ethyl 3-(1,3-benzothiazol-2-yl)-2-oxopropanoate	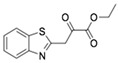	249.284	1.971	5	0	11.938 ± 1.782
**2D**	NSC56090 ZINC286970 (1S)-1-(1H-benzimidazol-2-yl)pentane-1,2,3,4,5-pentol	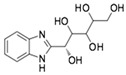	268.269	−1.329	6	6	48.107 ± 12.078
**2E**	NSC56093 ZINC4533419 (1*R*)-1-(1*H*-benzimidazol-2-yl)hexane-1,2,3,4,5,6-hexol	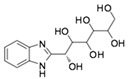	298.295	−1.968	7	7	46.108 ± 26.869
**2F**	NSC20641 ZINC4692139 *N*,*N*-dicyclohexyl-2,3,4,5,6-pentahydroxyhexanamide	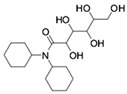	359.463	−0.084	6	5	44.509 ± 10.951

## Abbreviations

ZIKV	Zika virus;
HCV	Hepatitis C Virus;
DENV	Dengue virus;
WNV	West Nile virus;
JEV	Japanese Encephalitis Virus;
DAA	direct-acting antiviral agents;
NSP	non-structural protein;
NS3	non-structural protein 3;
NS3PRO	non-structural protein 3 protease;
NS3HEL	non-structural protein 3 helicase;
ssRNA	single-stranded RNA;
NTP	nucleoside triphosphate;
PDB	Protein Data Bank;
NCI	National Cancer Institute;
SBDD	Structure-based drug discovery;
SAR	Structure-activity relationship;
SFXC	Surflex-Dock algorithm;
